# Nurse practitioner led model of after-hours emergency care in an Australian rural urgent care Centre: health service stakeholder perceptions

**DOI:** 10.1186/s12913-021-06864-9

**Published:** 2021-08-15

**Authors:** Elena Wilson, Lisa C. Hanson, Kathleen E. Tori, Byron M. Perrin

**Affiliations:** 1 La Trobe Rural Health School, College of Science, Health and Engineering, La Trobe University, PO Box 199, Bendigo, Victoria 3552 Australia; 2 La Trobe Rural Health School, Violet Vines Marshman Centre for Rural Health Research, La Trobe University, PO Box 199, Bendigo, Victoria 3552 Australia; 3 School of Nursing, College of Health and Medicine, University of Tasmania, Locked Bag 1351, Launceston, Tasmania 7250 Australia

**Keywords:** NPs, Urgent care centres, Rural health, Health care delivery, Models of practice

## Abstract

**Background:**

The challenges of providing and accessing quality health care in rural regions have long been identified. Innovative solutions are not only required but are also vital if effective, timely and equitable access to sustainable health care in rural communities is to be realised. Despite trial implementation of some alternative models of health care delivery, not all have been evaluated and their impacts are not well understood. The aim of this study was to explore the views of staff and stakeholders of a rural health service in relation to the implementation of an after-hours nurse practitioner model of health care delivery in its Urgent Care Centre.

**Methods:**

This qualitative study included semi-structured individual and group interviews with professional stakeholders of a rural health service in Victoria, Australia and included hospital managers and hospital staff who worked directly or indirectly with the after-hours NPs in addition to local GPs, GP practice nurses, and paramedics. Thematic analysis was used to generate key themes from the data.

**Results:**

Four themes emerged from the data analysis: transition to change; acceptance of the after-hours nurse practitioner role; workforce sustainability; and rural context.

**Conclusions:**

This study suggests that the nurse practitioner-led model is valued by rural health practitioners and could reduce the burden of excessive after-hour on-call duties for rural GPs while improving access to quality health care for community members. As pressure on rural urgent care centres further intensifies with the presence of the COVID-19 pandemic, serious consideration of the nurse practitioner-led model is recommended as a desirable and effective alternative.

## Background

The challenges of accessing quality health care in rural regions have long been identified and include a lack of appropriate and sustainable community planning and infrastructure, increased disease chronicity and a shortage of highly qualified rural health service practitioners including allied health professionals and limited emergency care resources [1]. In rural Australia, urgent care centres (UCCs) provide varying levels of health care, including emergency services for rural communities. The centres provide care for patients presenting with minor injuries and illnesses, with provisions for initial resuscitation, limited life support and stabilisation for patients in a critical condition in preparation for transfer to a larger facility [2]. Unlike metropolitan and regional emergency departments (ED), rural UCCs are not resourced to provide ongoing definitive care for seriously ill patients.

There are many models for operating an UCC [2], with local General Practitioners (GPs) commonly responsible for medical responses in the centres. Although not a requirement, some GPs may have undertaken additional procedural skills education and training in areas such as surgery, anaesthetics, obstetrics, and emergency medicine [3–5]. A GP will often combine covering medical presentations in the urgent care centre with their private practice clinic. The provision of on-call after-hours medical services in addition to a typical GP working week is a major burden on work-life balance, is a known barrier for recruiting GPs to rural communities, and a reason for leaving rural communities [6–8]. These rural demands challenge the sustainability of the GP-led mode of care in rural areas.

An alternative and innovative model to address community and workforce needs and respond to the pressures of unmet health services in rural communities is the after-hours nurse practitioner (NP)-led service [9]. NPs have the capacity and clinical capability to support health care delivery services in geographically isolated communities and readily demonstrate a cost effective, safe, and competent alternate model of care [10–12]. NPs have completed additional training and have the necessary clinical skills and competencies to lead the provision of emergency health care in rural UCCs autonomously [2]. This includes the capability to assess, diagnose and treat; initiate referrals to other health care workers; request and interpret diagnostic tests; prescribe and review medications and discharge [12–14]. These skills and competencies are important in the delivery of safe and effective health care in a rural UCC, such as timely assessment and initiation of chest pain management [15] and the clinical capability to see and treat all presentations from mild through to high acuity [11]. Despite being introduced in Australia in 2000, the NP-led model of care has yet to realise its full potential as a legitimate option for addressing workforce needs in rural health care delivery [16–18].

In 2017, in response to after-hours medical workforce shortages and significant demand for after-hours urgent care, a small rural health service in Victoria introduced an after-hours NP-led model of care to its urgent care centre. After-hours NPs were contracted to provide health care provision over weekend periods from 8 pm Fridays through to 8 am Mondays. This service was provided by NP subcontractors from an external company. The NPs provided services to the UCC and as required, the acute ward and the aged care areas of the health service. Aside from the after-hours role, the NPs who provided this service did not hold other positions within the healthcare services. For the after-hours period, a GP was available to provide back-up assistance but were not required to be present in the UCC. The subcontracted NPs were supported by staff who were employed directly by the health service including registered nurses (RNs) and radiologists. During this period, a person who presented to the UCC was triaged by the RN, who then contacted the NP. The NP was responsible for the assessment, requesting any diagnostic procedures, initiating any intervention and deciding on whether the person required admission to the hospital for ongoing care, transfer to another health service or for discharge to their home with follow-up outpatient health care services. This research aims to explore the perceptions of rural health facility stakeholders in relation to the implementation of the nurse practitioner model of health care delivery in its urgent care centre in the after-hours environment.

## Methods

A qualitative design using individual or group interviews was used to address the aims of this research.

### Sample

Stakeholders were invited to take part in face to face or online semi-structured interviews or group interviews [19]. Using purposive sampling, 19 participants were recruited. Recruitment flyers were displayed in common areas at the hospital and distributed through an internal mail system. Recruitment occurred via an information stand at the organisation attended by two researchers. All stakeholders who responded to the recruitment flyer met the eligibility criteria. Group interview was offered for efficiency to participants with limited time availability.

Individuals were eligible to participate in the interviews if they were professional stakeholders of the afterhours NP-led UCC services. Professional stakeholders internal to the organisation included hospital managers and hospital staff who worked directly or indirectly with the after-hours NPs and professional stakeholders external to the organisation included local GPs, GP practice nurses, and paramedics.

As the research aimed to explore perceptions of professional stakeholders only, patients were not included in the sample. Informed consent was obtained from all participants. No participants were under the age of 18.

### Data collection: interviews

Sixteen semi-structured individual interviews and one group interview with three participants were completed between September and October 2018. Interviews were conducted by two researchers (EW and LH). Guiding questions explored perceptions of the impact of the after-hours weekend NP led service. The guiding questions varied slightly according to participant group, as illustrated in Table 1. Data collection continued until data saturation was reached [20].

**Table 1:** Guiding questions of semi-structured interviews and group interview

Common Guiding Questions for all participants	
What do you understand is the role of the NP for the urgent care centre? What do you see as the benefits of having a NP for the after-hours health care at the health service? What do you see as the enablers of the role of the after-hours NP at the health service? What do you see as the challenges of the role of the after-hours NP at the health service? Do you think the after-hours NP role offers a cost-efficient alternative to general practitioners? Do you think there are any benefits for people receiving a service from an after-hours nurse practitioner compared to general practitioners? To what extent do you believe that the after-hours NP role at the health service affects the perception of quality of health care provided to the community? Do you believe there is a future for the after-hours NP role at the health service and if yes, what, if any changes would you suggest to improve and sustain it as an ongoing service? Is there anything else that you wish to discuss that is related to the implementation of NP model for the health service	
**Additional questions**	
**Stakeholders with Management Role**	**Clinical Stakeholders without Management Role**
What do you see as the limitations of having a NP for the after-hours urgent care at the health service? How does the role of the after-hours NP sit within your organisation? Do you have experience managing other NP roles and in what way are they the same or different? Do you believe that the NPs were operating safely and within their scope of practice?	How did you find out about the after-hours NP role at the health service? Have you had reason to engage with the after-hours NP in your role at the health service? What are your experiences of working with the after-hours NP at the health service? What do you see as the limitations of having a NP for the after-hours urgent care at the health service? Do you believe that the NPs were operating safely and within their scope of practice?

### Data analysis

All individual and group interviews were audio recorded and transcribed verbatim. Transcriptions were transferred to NVivo12 data management software (QSR International 2018). The thematic analysis of data was led by the first author (EW) including coding of data into categories and sub-categories generating codes based on apparent thematic relationships and significant words and phrases [21]. Codes and themes were reviewed by the second author (LH). Conflicts were agreed by consensus and final themes confirmed by all authors. This process enabled ideas to be discerned that were central to understanding how staff and stakeholders of a rural health service perceived the implementation of an after-hours nurse practitioner model of health care delivery in its UCC.

## Results

Four themes emerged: transition to change; acceptance of the after-hours nurse practitioner role; workforce sustainability; and rural context.

### Transition to change

Participants revealed that a period of change had occurred during which clinical staff, stakeholders, and the community were adapting to the presence of after-hours NPs in the urgent care setting. From early challenges including reluctance to change, communication issues, and staff tensions, participants described a transition that led to some being resistant, some ambivalent and some embracing change.

#### Reluctance to change

Transition to change was often expressed by participants in terms of reluctance to change. Some members of the community had been seeing “*the same GPs for 30 years*” and wished to continue doing so.

“There is still a real perception that [they] should see the doctor as well. Still that stigma, that you are just a nurse, when is the doctor going to come and do the real stuff … ”

“ … at the start they were rather sceptical … a few of them were very upset in social media about GPs not being there or not being available to them readily - as readily as they had been”

#### Communication issues

Even though clinical staff regarded the reluctance for change as “*just teething problems*” that would “*resolve over time*”, the challenges of this transition were attributed by some participants to the lack of communication about the introduction of the model. External stakeholders reported they were not made aware of the changes and their potential impact before implementation of the NP model. In addition to improving communication about the NP model within the hospital, participants expressed the need to increase awareness about its scope and priorities for service provision. Some participants spoke about initial hesitation by patients to see NPs instead of GPs and recommended greater community education about abilities and scope of practice of NPs and the model as a safe and effective, no-cost service.

“It was implemented before we were informed officially, and we were told that it was happening post the implementation of the role - by the local paper, not by the hospital directly”.

“I found out just from talking to the nurses up at urgent care that it was being implemented but I hadn’t heard anything official *…* it was sort of like we’d just rock up and find out they were there”.

Several participants believed that there had been an absence of any education to build an understanding of the after-hours nurse practitioner role and its limits leading to confusion about the NP role.

“We are still, from a nurse perspective … trying to work out what their actual role is … they are not just nurses … but [we are] not fully aware of the scope of practice that after-hours NPs have.”

Or not fully aware of their capabilities or … how educated or how well trained they are … [so] we are not sure if we refer to after-hours NPs or GPs …” .

“My understanding is [after-hours NPs] take place of a GP.”

#### Staff tensions

Although the after-hours NPs were well received in the health service’s after-hours UCC, tensions between after-hours NPs and RNs were described particularly in relation to professional identity and leadership. Some RNs perceived “*a lot of just bitchy kind of belittling stuff*” often associated with delineation of roles:

“There are particular nurses who get their noses out of joint, believe the after-hours NPs are still a nurse and try to drag [them] back to, well you can change the bed, you can do that, or I’m not going to take orders from you, you’re a nurse, who do you think you are.”

Conversely, there were others who saw after-hours NPs as providing a “*real bonus*” as they were able to take decision-making out of the hands of the RNs:

“By having someone higher trained we can say what do you think and pass that on … and we've met so many who are very well educated and [with] huge amount of knowledge that they can share with us, and huge amount of experience that they've had that they share*.”*

### Acceptance of the after-hours nurse practitioner role

Despite the challenges presented, most participants described a transition towards acceptance of the model, adopting a welcoming and grateful approach. People were thought to have “gone from being very sceptical and not happy, to anybody that has dealt with them has been very happy … and that goes around town very quickly”. With “… six and a half thousand presentations a year” the health service had not received any complaints about the after-hours NPs. Participants reported always trying to “make them feel welcome … orientate them to the town, … and thanking them for providing that service to us because it is a big thing.” The model was commonly thought to provide a positive and appropriate solution for the organisation*.*

“I think the overall principal is fantastic and when you've got a great after-hours nurse practitioner, the world is a happy place.”

The after-hours nurse practitioner role emerged as multi-faceted, largely understood by participants as bridging a gap between GPs and RNs yet also seen to combine such elements as advanced clinical skills, holistic patient management, and empowerment and education of nurses.

Overwhelmingly, participants who were health service staff perceived the after-hours nurse practitioner role to be an “*on-call service that could bridge the gap between GPs and RNs*”. They were described as “*the same as a doctor but a little bit different* [and] *can step into an emergency*” when there is “*no on-call GP to assist and support*” the GP. However, despite the similarities, after-hours NPs were considered to work differently to the GPs with some perceiving limitations of the NP role such as:

“they can't see a large number of patients within a period of time. They are certainly not as efficient in, call it triaging, a large number of patients in a short period of time. They spend a lot more [time] on investigations”

In relation to their clinical skills, NPs were considered “*very knowledgeable* [and] *really highly skilled clinicians* [who have] *wonderful insight* [and] *do an exceptionally good job*”. The after-hours NPs’ skillset was described as “*phenomenal*”, “*fantastic*”, and “*bloody brilliant*”. Respect for the after-hours nurse practitioner role was also evident in statements about their clinical skills:

“I sort of admire them for their skill set and scope and the extra knowledge that they bring into the emergency nursing environment … they always say they are not doctors, but they still do have the clinical skills to look after the majority of cases that front to the UCC”.

Despite the high regard for the NPs’ advanced clinical skills, some limitations relating to their scope of practice were reported. Clinical staff cited limitations relating to mental health care, regarding “*sectioning and things like that*” in which case staff “*would bring the doctor in*”. (Sectioning refers to the involuntary admission and treatment of patients for mental health care). When patients required treatment that was not accessible to the after-hours nurse practitioner due to Medicare limitations examinations were not possible without a GP. Participants expressed recognition that the after-hours NPs were aware of the limitations of their scope of practice and worked within them:

“the after-hours nurse practitioner knows their limits, so they don’t muck around, beat around the bush, and sit on it for 5 hours, … they are quite decisive in their knowledge base and they know when they have reached the limit of their scope of practice”

After-hours NPs were thought to have a more contemporary approach to health care based on evidence that “*is more up to date*” and delivered more holistic care to patients than the GPs. Holistic patient management meant they provided explanations to patients and staff while it was perceived that “*the doctor generally won’t*”. Advanced clinical skills and holistic care were thought to be attributes that rendered the after-hours nurse practitioner “*streets ahead* [and] *more capable*” than GPs, some commenting on the need for improved knowledge for those who had been practicing a very long time. NPs were admired for “not only the acute presentation, but the ongoing care, like long term. I’ve heard after-hours NPs have conversations about six months to twelve months care for [a] patient based on their concerns”.

The after-hours NPs were seen as coming into the health service with a new approach, which had the effect of “*empowering the nurses that are working alongside*” them while also fulfilling an educational role for the health service staff:

“Look I think there's probably a learning thing for our staff. They're well qualified, and actually, you know, running some in-services … it's a great benefit.”

“They explain what is going on. To the staff and patients.”

There was a sense that the RNs were not usually encouraged to give an opinion:

“No one really takes into account their opinions on patients … the nurses have never really been asked [their opinion] before”.

Their educational role extended to discussing policies and procedures and how to improve them thereby further building capacity of the nursing staff.

### Workforce sustainability

Participants identified challenges to the sustainability of a rural, after-hours, urgent care workforce that stem largely from factors such as burden on the GP, the changing nature of the workforce, and cost, all of which contribute to unequal supply and demand of practitioner workforce.

“This hospital's UCC sees 6000 patients per year … without any fulltime doctors at the hospital … and it just became impossible with the number of practitioners in town to actually service the urgent care on a 24/7 basis”

#### GP burden

This demand on GP service provision was thought to place a burden on the rural GP role, which was itself considered a “*finite resource”* that had become strained. Stakeholders expressed concern for the wellbeing and work-life imbalance experienced by rural GPs describing them as “*ageing, … overworked, … and fatigued*” and needing to “*get some rest*”. Concerns extended to the ability of GPs to provide quality care, the need to update their knowledge and the risk of the community being left without a GP.

“… when the one GP goes on leave or as we know in this local area it does happen that a husband and wife team will go on leave and therefore a small town’s left without a GP and public hospital without a GP”.

Stakeholders expressed concern that the capacity of one or two GPs to sustain quality care to the community and hospital 7 days a week was strained and feared that GPs would leave the community exacerbating challenges of recruiting medical staff to rural areas.

The NP-led model for after-hours urgent care was a new model of care designed to alleviate the workload burden of rural GPs and improve recruitment and retention of rural GPs. Acceptance of the new model was influenced by both the attributes of the individual NPs and the organisational support and leadership, specifically, the “*years of experience*” of the NPs and the “*proactive*” support of the health service’s management team.

“… the main thing is the reduction of the workload of general practitioners which meant that we were able to retain general practitioners in our environment for longer.”

Participants also recognised that the after-hours workload was transferred from one group to another group. There was concern about potential impacts on the health and well-being of the contracted NPs.

“… there is the potential to drive the after-hours nurse practitioner into the ground as well … there is a high risk of burnout there too. It is a really busy service.”

Participants remarked on the challenges of working long hours in one block which were incompatible with rostering *“one after-hours nurse practitioner for the entire weekend not realising the workload of the hospital*.”

#### Changing workforce

The changing nature of the workforce was evident from the observations made by participants about recruitment and retention of practitioners. Although participants saw their GP workforce as ageing, they believed that:

“… older GPs seem to accept that long hours working model, where the newer GPs that come don’t, they don’t seem as willing to not have any life at all.”

Having lost several local GPs, participants commented that the main reason practitioners gave for leaving town was the need for covering after-hours urgent care, and “*[wanting] a better work-life balance*”. Similarly, “*great difficulty*” had been experienced recruiting doctors “*because when they asked about the afterhours load, they would just say ‘well thank you very much but I’m not going to do that*’”. The after-hours nurse practitioner model was, therefore, seen as “*a great benefit for the [recruitment and] retention of general practitioners*” even though participants accepted that “*really the future of any program is based around funding*”.

#### Cost

Participants commented that the cost of the after-hours nurse practitioner model, when a contracted service was used, was more cost effective than the traditional GP-led service. This was despite the GPs still being on-call. Compared to GPs, NPs were thought to produce cost savings through better patient flow and staff engagement. An example offered was that NPs were more likely to assess patients within the recommended timeframes and admit patients to the hospital if they required treatment and the hospital would attract hospital inpatient funding and “… can almost hire three of them for the price of one GP, there is definitely a lot of benefits.”

Despite the perceived cost benefits, some participants viewed the cost difference between staffed urgent care and on-call NPs to be restrictive as the on-call service did not attract Medicare funding for the health service.

“Because it’s not funded it’s absorbing a very large, very significant portion of the hospital’s budget to pay for their services … working for the hospital they get salary and they can’t charge Medicare fees, so there’s no offset to costs.”

NPs were commended for providing thorough assessment but were thought to order more tests such as pathology screening and radiography assessment and utilise ambulance services more often, thereby increasing the cost of care. This cost was, however, thought to be offset by the benefits in the quality of patient care.

### Rural context

Participants emphasised the uniqueness of rural health practices compared with those in metropolitan areas. A common explanation was that working in a small rural health service UCC was very different to working in a metropolitan emergency department and could pose challenges for non-rural after-hours NPs rostered at the health service particularly in relation to isolation and service system capacity.

Isolation was considered the biggest challenge especially for after-hours NPs without previous experience working in isolation in a small town.

“… they’re coming to do their shifts from outside areas and they might not have a good understanding of the isolation of the area and that kind of thing unless they’ve done a shift here before. And they may not necessarily understand what’s involved before they get here so it might be a bit of a surprise.”

There was a perception that some NPs with primarily metropolitan experience over-reported capabilities and did not appreciate the limitations associated with service system capacity, or provisions for ongoing life-support measures and that some tried to replicate a metropolitan hospital emergency department in the rural facility.

“… a lot of them are just not used to working in a small town where perhaps they only have them and a couple of nurses, and it is them making the final call and the final decision ... if you’re here you’re kind of it”.

Service system capacity was described as a limitation of the rural location of the health service. Examples given by participants related to limited facilities and the ongoing issue of weekend rostering for after-hours urgent care.

“We might not have as many facilities ... access to fairly extensive pathology isn’t necessarily available here on the weekends … and X-Ray and other ultrasound is not necessarily as quickly available.”

“We’re a small town so we don’t have an enormous emergency department and doctors on site … rostering has been and perhaps still is an issue for them moving forward … especially on weekends when they get a lot of self-presentations.”

Generally, it was thought that after-hours NPs with previous experience in rural UCCs fitted in more easily, had better awareness of “*the limitations of regional areas as a whole in terms of ongoing care*” and were “*better at seeing that this is how it is and getting on with it*”. On a practical level staff facilitated the after-hours nurse practitioner role by making sure they were “*documenting everything, providing them with a whole picture, not just a snippet of what’s going on*”. However, while participants acknowledged that they respected and valued the after-hours NPs as highly trained practitioners in emergency and critical care, there was a perception that a thorough competency checklist or credentialling needed to be completed by the hospital to overcome some rural challenges and meet the expectations and requirements for working in rural and isolated practice.

## Discussion

Themes identified in this study converge to highlight the multifaceted implications of introducing a NP-led model of after-hours urgent care in a rural health service.

The health service in this study is one of the many in rural and remote regions coping with cyclic challenges of a declining rural GP workforce, difficulty recruiting and retaining a nursing workforce, and the inequity of access to urgent health care for rural communities [11, 22]. While affirming these well recognised problems, this study reveals the potential of a NP-led model of after-hours urgent care for circumventing them while also adding value through empowering education and mentoring of nursing staff.

Determined to address the large number of emergency admissions in its after-hours UCC, the rural health service in this study sought to enhance its existing services by introducing the NP led model. What the health service achieved, however, was much more. Decision making capacity including the ability to assess, treat and manage varying acuity of clinical presentations is well within the realm of the NP role if adequately prepared for such contingencies. Several studies [23–25] have demonstrated that advanced practice nurses, such as NPs, if appropriately educated and trained, can provide health care at the same level as their medical colleagues. Described as “leaders in dynamic, unpredictable settings that required their sophisticated nursing knowledge and expertise” [26] NPs were viewed, in this study, to have demonstrated leadership qualities through their mentoring and education of registered nurses. Their commitment to empowerment and building capacity in others through a more contemporary and holistic approach to health care based on up-to-date evidence was a welcome additional benefit for the health service’s existing workforce which consequently held them in high regard.

Despite these benefits, some caution is justified for introducing this model, particularly in relation to rural preparedness, transfer of burden, communication, and cost considerations. Ensuring NPs are appropriately educated for entering the rural urgent care environment would prepare them for coping with the complexities of the rural context, including for integrating with health service staff and stakeholders in those rural settings [27, 28]. NPs who have practiced predominantly in metropolitan emergency departments may be challenged by professional and geographic isolation [29]. Although independent practitioners in their own right, limited service system capacity, can mean that NPs ultimately make final decisions about clinical care independently, often in the absence of diagnostic services such as x-ray and pathology. NPs new to the rural urgent care environment may not be prepared for practicing with limited resources.

Cost savings were thought to be produced through improved patient flow and staff engagement [30] compared to a GP-led model. However, variations to cost efficiency may result from jurisdictional regulations relating to medical rebates and health service funding which may also be dependent on types of employment arrangement.

Increasing demands on rural UCCs have been exacerbated by a shifting GP workforce. Burden on healthcare professionals can lead to what is sometimes referred to as burnout syndrome, which has also been associated with an increased rate of turnover with healthcare professionals leaving their fields to seek alternative professions [31, 32]. A changing of the guard from retiring GPs to a new generation of GPs has led to a shortage in the rural GP workforce and highlights the expectation of work-life balance that makes rural practice unsustainable for some [33, 34]. This challenge for rural practice calls for more than short-term incentives for medical graduates to work in rural communities [35]. The presence of NPs has potential to prolong GP retention and positively impact GP recruitment [7] thereby enabling patients to receive timely care in their local community and limit the need for transfers out of town [7, 36]. In these ways, NPs can add value to the health service and the community it serves. While it is recognised that rural emergency doctors are generally older than their urban counterparts and numbers are decreasing [37], it is similarly true that the nursing workforce is also ageing, and in some countries such as Australia and New Zealand this includes NPs [38]. The burden associated with rural GPs therefore, risks being transferred to NPs if wider workforce implications are not factored into implementation of NP-led models in rural and remote UCCs.

Implementing a new model of health care delivery requires open and transparent communication. A strategic and collaborative approach between the health service, stakeholders, patients, and community members is necessary for developing a timely communication plan to introduce the NP-led model to influence attitudes within these groups, particularly relating to trust [39]. A planned communication strategy could avoid the element of surprise and any misunderstandings or assumptions about delineation of roles and processes during the transition period for health service staff and for stakeholders [40].

Addressing access inequity for rural and remote health services continues to be a policy priority in Australia and internationally [41, 42]. In Australia more than 7 million people, 28% of the population, live in rural and remote communities and continue to experience poorer health outcomes and poorer access to and use of health services than people living in metropolitan areas [1, 43]. Similar health disparities occur in other developed countries such as Canada, where 25% of people live in rural and remote areas [44], and approximately 30% in NZ [45]. The problem of uneven distribution of care between rural and metropolitan communities must be addressed through innovative models of health care delivery. The NP-led model of after-hours urgent care offers one such model for addressing the emergency care component specifically.

The role of a NP encompasses a scope of practice which overlaps with that of medicine yet remains within the nursing practice domains, filling an essential practice role, periodically referred to as the third space [46]. NPs do not practice medicine however are well placed to see patients across the full continuum of care and have proven valuable in providing care in both acute and primary health care roles [47]. The reframing of professional boundaries between nursing and medicine is an established phenomenon that features nurses taking on more of the tasks that previously belonged to the medical profession [23] and has shown significant growth of the NP role in Australia, in both endorsement numbers and specialty areas [9]. Although the role was originally intended for underserviced areas of primary and community health, most NPs practice within hospital acute care sectors [22]. The NP-led model offers a logical response to the pressures of unmet health service need in rural areas [48].

Increasing demands on rural health services threaten their ability to deliver sustainable, quality urgent care. Rural communities world-wide continue to endure not only the additional demands imposed by the 2020–2021 COVID-19 pandemic [49, 50], but also destructive natural disasters [51]: in Australia these have taken the form of pervasive bush fires and floods [52]. These unrelenting demands continue to add further strain to an already fragile rural health workforce [53]. Hence, the significance of this study is amplified as disaster preparedness adds another layer of complexity for rural emergency departments struggling to remain resilient. Possessing the agility to respond to these challenges is necessary if inequity of service provision to already vulnerable populations is to be diminished: the NP-led model for after-hours urgent care can be leveraged to offer health services that agility [54] making it a desirable and necessary alternative.

As workforce and practice challenges persist, the NP-led model can offer benefits to both rural after-hours UCCs and the communities they serve. Health services considering a NP-led urgent care model, however, need to be aware of potential barriers to a smooth workforce transition and act to minimise them. The NP-led model does not eliminate the need for GPs but adds a much-needed injection of clinical expertise with potential for development of skills and confidence for existing nursing staff thereby positively impacting the quality of health care delivery.

### Limitations

A purposive sample has limitations as it may not be representative of the health care practitioners in general. Guiding questions may have influenced some responses. The findings of this study cannot be generalised to other rural health services as it explores the perceptions of health service staff and stakeholders of one rural health service. Despite this focus, authors believe this study provides meaningful insight to issues that may be present in other rural urgent care settings. The perceptions and experiences of rural urgent care users were not investigated in this study. This would be an important consideration for future research in this area.

### Recommendations

Key recommendations from this research include: to continue to develop, monitor and refine the NP-led model for rural UCCs so that rural health services have access to a sustainable workforce that is prepared for nuances of working in a rural context; to develop a governance strategy or steering committee to guide and support the NP model thereby assisting with user acceptance of the model and mitigation of risk; to develop and implement a communication plan that raises awareness in rural communities of the NP role in the after-hours UCCs; and to provide education and documentation that clarifies the delineation of roles and responsibilities of clinical staff to help dispel challenges in adapting to change. A final recommendation relates to the cost effectiveness of the model alongside the perceived problems with the lack of Medicare. It is recommended that further research is done for a better understanding and that sustainable models of funding are sourced to enable the ongoing role of a NP-led model of health care delivery in UCCs.

## Conclusion

The opinions of participants suggest that the nurse practitioner-led model is valued by rural health practitioners and could reduce the burden of excessive after-hour on-call duties for rural GPs while improving access to quality health care for community members. As pressure on rural UCCs further intensifies with the presence of the COVID-19 pandemic and threats of future natural disasters, serious consideration of the nurse practitioner-led model is recommended as a desirable and effective model.

## Data Availability

The datasets generated and analysed during the current study are not publicly available to protect participant privacy but are available from the corresponding author on reasonable request.

## Abbreviations

ED: Emergency Department
GP: General practitioner
GPs: General practitioners
NP: Nurse practitioner
NPs: Nurse practitioners
RN: Registered nurse
RNs: Registered nurses
UCC: Urgent Care Centre
UCCs: Urgent Care Centres
